# Oxidative Stress and Blood Parameters During Shearing in Sheep Treated with Melatonin

**DOI:** 10.3390/ani16111699

**Published:** 2026-06-01

**Authors:** Vincenzo Carcangiu, Sebastiano Luridiana, Raffaella Cocco, Othmane Trimasse, Imane Bennaghmouch, Mortadha Ouadday, Maria Consuelo Mura

**Affiliations:** Department of Veterinary Medicine, University of Sassari, Via Vienna 2, 07100 Sassari, Italy; vcarcangiu@uniss.it (V.C.); sluridiana@uniss.it (S.L.); rafco@uniss.it (R.C.) o.trimasse@studenti.uniss.it (O.T.); i.bennaghmouch@studenti.uniss.it (I.B.); mortadha.ouadday@gmail.com (M.O.)

**Keywords:** sheep, melatonin, shearing, stress, oxidative stress

## Abstract

Shearing is a stressful procedure for sheep, combining isolation, restraint, and the mechanical action of shearing, which activates the hypothalamic–pituitary–adrenal axis and induces oxidative stress. This study investigated whether melatonin—a pleiotropic hormone with well-documented antioxidant properties—administration could modulate the stress response and oxidative stress in Sarda sheep during shearing. Forty lactating ewes (aged 3–5 years, mean body weight 41 ± 1.1 kg) were randomly assigned to four groups (*n* = 10 each): two groups received a subcutaneous melatonin implant (18 mg); two remained untreated as controls. Within each category, one group was shorn and the other subjected only to restraint. Blood samples were collected before, during, and after shearing to measure cortisol, glucose, reactive oxygen metabolites (ROMs), biological antioxidant potential (BAP), and oxidative stress index (OSI). Procedures elevated cortisol, glucose, ROMs, and OSI in all groups, but melatonin treatment significantly reduced these parameters and increased BAP relative to untreated animals at all sampling points (*p* < 0.05). No significant differences were observed between shorn and unshorn animals within the same treatment, suggesting that the handling, restraint, and isolation associated with the shearing procedure represent the major sources of stress, rather than the mechanical act of shearing itself. In conclusion, melatonin administration blunts the stress response and reduces oxidative stress in sheep during routine shearing-related handling procedures, suggesting its potential as a practical tool to improve animal welfare during routine management practices.

## Simple Summary

Shearing is a necessary practice for sheep welfare, but it causes stress due to restraint, isolation, and the shearing procedure itself. Stress can lead to the production of harmful free radicals, a condition known as oxidative stress, which may negatively affect animal health. Melatonin is a natural hormone known for its antioxidant properties and its ability to regulate sleep and seasonal rhythms. This study investigated whether treating sheep with melatonin before shearing could reduce their stress response and oxidative stress. Forty ewes were divided into four groups: some received a melatonin implant and were either shorn or left unshorn, while others remained untreated and were either shorn or left unshorn. Blood samples were collected before, during, and after shearing to measure stress hormones, energy levels, and markers of oxidative stress. The results showed that melatonin-treated sheep had lower stress hormone levels and better antioxidant capacity compared to untreated animals, both during and after shearing. These findings suggest that melatonin administration can help sheep cope with the stress of shearing, reducing physiological indicators associated with stress during this routine management practice. Melatonin administration may represent a useful approach to modulating physiological stress responses during routine handling procedures in sheep.

## 1. Introduction

Many management practices in farmed animals, although necessary, elicit endocrine and metabolic responses collectively referred to as stress [1]. Procedures such as transport, weaning, hoof care, veterinary interventions (treatments, vaccinations, blood sampling, etc.), shearing, and isolation, while essential for animal management and welfare, can disrupt physiological homeostasis [2,3]. In addition, environmental factors such as extreme temperatures and malnutrition may also induce stress [4]. The magnitude of the stress response depends on several factors, including individual sensitivity, prior exposure to the stressor and its intensity [5], as well as social hierarchy and sex, with males generally being reported to be less sensitive [6,7].

The stress response primarily involves activation of the sympathetic nervous system and the hypothalamic–pituitary–adrenal (HPA) axis, leading to the release of catecholamines and glucocorticoids, respectively [8]. These hormones enhance alertness and enable animals to respond to environmental challenges, thereby contributing to the maintenance of homeostasis [9]. They also promote increases in blood glucose through the mobilization of both carbohydrate and non-carbohydrate substrates (e.g., proteins), as well as elevations in non-esterified fatty acids (NEFAs) resulting from lipid mobilization [10]. Collectively, these metabolic adjustments increase energy availability for the brain and muscles, facilitating an effective behavioral response to stress.

Stress is also associated with increased production of reactive oxygen species (ROS), leading to oxidative stress [11]. This condition arises from an imbalance between pro-oxidant and antioxidant systems at the cellular level, favoring oxidants [12]. Oxidative stress may occur when antioxidant defenses are overwhelmed by increased oxidant production or reduced antioxidant availability [13]. If not adequately counteracted, this imbalance can result in cellular damage [14] and contribute to the development of degenerative diseases [15,16]. In farm animals, oxidative stress is implicated in numerous pathological conditions affecting productivity, reproduction, and overall welfare [17].

Melatonin is primarily synthesized and secreted by the pineal gland during the dark phase, under the control of the suprachiasmatic nucleus, which regulates circadian and circannual rhythms [18,19,20,21]. Its secretion is also influenced by stress, with effects varying according to the species’ diurnal or nocturnal activity patterns [22,23,24]. Melatonin modulates behavioral, endocrine, and neurobiological responses to stress, and its administration has been shown to attenuate stress-induced alterations [25,26,27]. However, most studies have been conducted in nocturnal species (e.g., rats) under chronic stress conditions, whereas limited data are available for diurnal animals exposed to acute stress.

Sheep are diurnal mammals, and the effects of melatonin have been extensively studied, mainly regarding seasonal rhythms and reproduction [21], on milk production, pregnancy, and lamb birth weight.

Melatonin is a pleiotropic hormone and has a direct effect in the antioxidant cascade, as it acts, through its metabolites (e.g., cyclic 3-hydroxymelatonin), as a scavenger of free radicals (ROS/RNS) [28].

The initial derivative of melatonin is cyclic 3-hydroxymelatonin (c3OHM), which functions as an acceptor of free radicals; each melatonin molecule can neutralize up to 10 ROS/RNS [29]. This unique property of melatonin makes it highly effective in combating oxidative stress. Melatonin is produced in many cells and its synthesis can be upregulated under conditions that increase oxidative stress in mammals [30]. Melatonin also indirectly stimulates the activities of a variety of antioxidant enzymes including manganese and copper-zinc superoxide dismutase, glutathione peroxidase and reductase, and glutamylcysteine ligase [31].

Several studies on sheep have been conducted on the response to stress determined by different environmental factors. Among these, shearing is a procedure that causes severe stress to the animal as it is a combination of various actions such as isolation, restraint, the action of the shears, etc. In the present study, shearing refers to a traditional manual method, which requires physical restraint of the animals and is therefore more time-consuming compared to mechanical clipping systems, where restraint is minimal and the procedure is faster. Furthermore, during shearing, it has been observed that sheep show a sharp increase in free radicals, compensated by the body’s endogenous production of antioxidants [11].

Given the limited number of studies investigating the effects of melatonin under acute stress conditions in sheep, further research is warranted. Therefore, the aim of the present study was to evaluate whether melatonin administration modulates cortisol secretion, selected metabolic parameters (including glucose and non-esterified fatty acids), and oxidative stress markers (pro-oxidant and antioxidant indices) in Sarda sheep exposed to shearing-related handling procedures, including restraint and isolation.

## 2. Materials and Methods

### 2.1. Animals

The study was conducted on a farm, located in Northern Sardinia (40°48′ N; 8°16′0 E, 69 m above sea level), which raised approximately 600 Sarda sheep. The animals were reared from birth under natural photoperiod. During the day the sheep grazed on legumes and gramineous grasses and at the time of milking they received 300 g of commercial concentrated feed per capita per day (20.4% crude protein and 12.5 MJ ME/kg of DM). During the night the sheep were penned and received hay (11.1% crude protein and 7.2 MJ ME/kg DM) and water ad libitum. For this study, 40 lactating ewes were selected. The animals were aged 4.1 ± 1.1 years, had a body condition score (BCS) 2.8 ± 0.5, and had lambed before December of the previous year. The enrolled ewes were clinically healthy with no evidence of disease and free from internal and external parasites. Traditionally, Sarda sheep are shorn once a year, in late spring, according to the traditional techniques of Sardinian sheep breeding. All 40 sheep chosen had been shorn in previous years and were familiar with the handling required for venipuncture.

### 2.2. Ethics Approval

All the animals used in this scientific research had National Health Veterinary Service care in accordance with the Animal Welfare Act. All procedures described in this study were conducted as part of routine farm management practices for dairy sheep, including shearing, restraint, and blood sampling. Nevertheless, because the data were collected during routine farm management in 2025, the study protocol was retrospectively reviewed and approved by the animal ethics committee of the University of Sassari (Protocol number: 27998), granted on 30 March 2026. The Committee confirmed that all procedures complied with animal welfare standards.

### 2.3. Experimental Design

On 10 May 2025, the 40 ewes were randomly distributed into four groups (A, B, C, and D) of 10 ewes each (*n* = 10), balanced based on lambing date, parity, and age. On the same day, groups A and B received a subcutaneous melatonin implant in the periauricular area, using the standard commercially available slow-release melatonin formulation for adult sheep (Melovine^®^, CEVA Salute Animale, Agrate Brianza, Italy), containing 18 mg of melatonin released according to the manufacturer’s specifications, while groups C and D remained untreated and served as controls. Shearing procedures and blood sampling were subsequently performed on 1 June 2025, corresponding to 22 days after implantation.

The animals were divided into the following experimental groups:

Group A (Mel+Shorn): 10 ewes treated with melatonin; they were separated from the flock, their feet were tied, and they were subsequently shorn.

Group B (Mel+Unshorn): 10 ewes treated with melatonin; they were separated from the flock, their feet were tied, and they were not shorn, but were kept tied and placed in lateral recumbency for the duration equivalent to shearing (approximately 15 min).

Group C (No Mel+Shorn): 10 ewes, untreated with melatonin; they were separated from the flock, their feet were tied and they were subsequently shorn.

Group D (No Mel+Unshorn): 10 ewes, untreated with melatonin; they were separated from the flock, their feet were tied, and they were not shorn but were kept tied and placed in the lateral recumbency for the duration equivalent to shearing (as B group).

All enrolled ewes were separated from the rest of the flock for a week in a covered enclosure where shearing was performed. Shearing was performed by hand using traditional shearing scissors, on 1 June 2025, in a 30 × 10 m enclosure (ambient temperature 28 ± 2 °C and relative humidity 58 ± 6%) divided into two sections by a partition that allowed the animals to see and hear each other. The groups were identified by a different colour mark on their rumps.

The entire procedure lasted approximately 15 min for each sheep, whether shorn or unshorn, and it took about 1.5 h to perform the treatments on all four groups.

### 2.4. Blood Sampling and Analysis

From each ewe, blood samples were collected via jugular venipuncture (20G needle) using tubes containing lithium heparin (Becton Dickinson, Plymouth, UK) at four time points: the day before shearing at 8:00 AM (T1); immediately after restraint and before the beginning of shearing at 8:00 AM (T2); immediately after completion of shearing (T3); and the day after shearing at 8:00 AM (T4). Sampling from the shorn and unshorn groups was carried out in the same way and at the same time. Blood was immediately centrifuged at 3000 rpm for 20 min at 4 °C, and the resulting plasma was frozen at −20 °C until analysis. Plasma cortisol levels were determined using an ELISA kit specific for ovine species (Bovine/Sheep Cortisol ELISA kit, Elabscience Biotechnology Inc., Kampenhout, Belgium) with an automatic reader (Sirio, SEAC, Florence, Italy). Both the intra- and inter-assay coefficients of variation were <10%.

Plasma glucose and triglycerides were analyzed using enzymatic colorimetric methods by commercial kits (Spinreact, Girona, Spain).

Plasma oxidants and antioxidant capacity were analysed with two commercial kits (Diacron, Grosseto, Italy) by determining reactive oxygen metabolites (ROMs), mainly hydroperoxides produced by the oxidation of biomolecules, and biological antioxidant potential (BAP), measuring the capacity of blood to reduce iron from ferric (Fe^3+^) to ferrous (Fe^2+^) form. The d-ROMs test was expressed in Carratelli units (U. Carr.), where 1 U. Carr. corresponds to 0.08 mg/100 mL H_2_O_2_. BAP was expressed in µmol/L of reduced iron. The oxidative stress index (OSI) was calculated using the formula (ROMs/BAP) × 100, proposed by Ranade et al. [32].

### 2.5. Statistical Analysis

All statistical analyses were performed using R (version 4.5.2; R Foundation for Statistical Computing, Vienna, Austria). Data were initially screened for normality using the Shapiro–Wilk test and for homogeneity of variances using Levene’s test. When necessary, variables were log-transformed to meet the assumptions of parametric analysis.

The experiment followed a 2 × 2 factorial design with melatonin treatment (melatonin vs. no melatonin) and shearing treatment (shorn vs. unshorn) as between-subject factors. Blood variables were measured repeatedly at four time points (T1, T2, T3, and T4); therefore, sampling time was included as a within-subject factor.

The effects of melatonin treatment, shearing treatment, time, and their interactions on plasma cortisol, glucose, triglycerides, reactive oxygen metabolites (ROMs), biological antioxidant potential (BAP), and oxidative stress index (OSI) were analysed using linear mixed-effects models based on the General Linear Mixed Model framework. Individual ewe was included as a random effect to account for repeated measurements within animals.

The statistical model used was as follows:

Y_ijkl_ = μ + M_i_ + S_j_ + T_k_ + M_i_S_j_ + M_i_T_k_ + S_i_T_k_ + M_i_S_j_T_k_ + A_l_ + e_ijkl_

Y_ijkl_ = the observed value of the dependent variable;

μ = overall mean;

M_i_ = fixed effect of melatonin treatment (i = melatonin, no melatonin);

S_j_ = fixed effect of shearing treatment (j = shorn, unshorn);

T_k_ = fixed effect of sampling time (k = T1–T4);

A_l_ = random effect of animal (l = 1–40);

e_ijkl_ = residual error.

Linear mixed models were fitted using the lme4 package [33], and significance of fixed effects was assessed using the lmerTest package [34]. When significant main effects or interactions were detected, pairwise comparisons were performed using Tukey-adjusted post hoc tests based on estimated marginal means implemented in the emmeans package [35].

Results are reported as mean ± standard deviation (s.d.). Differences were considered statistically significant at *p* < 0.05, while 0.05 ≤ *p* < 0.10 was considered a statistical tendency.

## 3. Results

The results indicate that several parameters analysed were affected by both the treatment and the experimental procedures.

### 3.1. Cortisol

Blood cortisol concentrations increased in all groups from the day before sampling (T1) to pre-shearing (T2) and at the end of shearing (T3). Values returned to pre-shearing levels at T4 (the day after the procedures), with levels comparable to T1. At all time points, cortisol concentrations were lower in melatonin-treated groups (A and B) compared to untreated groups (C and D) (*p* < 0.05). No differences were observed between Group A (melatonin-treated, shorn) and Group B (melatonin-treated, unshorn), nor between Group C (untreated, shorn) and Group D (untreated, unshorn). The lack of a significant interaction between melatonin treatment and shearing suggests that melatonin mainly modulates the overall stress response, rather than the component specifically associated with the mechanical action of shearing. Peak cortisol concentrations were observed at T3, approximately 15 min after the onset of the procedures, in all groups. No significant melatonin × shearing interaction was detected for any of the measured parameters (*p* > 0.05 for all) (Table 1).

### 3.2. Glucose

Glucose levels were higher in Groups C and D compared to Groups A and B at all sampling times (*p* = 0.04). The greatest increase in all four groups was observed at the third sampling (T3) in both shorn and unshorn animals (Table 2).

### 3.3. Triglycerides

Plasma triglyceride concentrations showed no variations throughout the observation period in any of the four groups (*p* = 0.12) (Table 3).

### 3.4. ROMs

Animals treated with melatonin showed lower ROM levels compared to untreated animals at all sampling times (*p* = 0.02). All groups showed an increase with the start of the shearing procedures, but Groups C and D exhibited the highest values (*p* = 0.03). Shorn and unshorn animals, whether treated or not, showed similar ROM levels (Table 4).

### 3.5. BAP

Plasma BAP concentrations were higher in treated animals compared to those untreated with melatonin at all sampling times (*p* = 0.01). All groups showed a decrease in BAP levels with the start of the shearing procedures (*p* = 0.03). Treated animals, both shorn and unshorn, showed no differences in BAP levels at any sampling time; the same trend was observed in untreated animals (Table 5).

### 3.6. OSI

OSI values were higher in untreated animals at all sampling times compared to treated ones (*p* = 0.03). In all groups, an increase in OSI values was observed with the start of the shearing procedures, with the highest values recorded at the second and third samplings (*p* < 0.05) (Table 6).

## 4. Discussion

Traditional shearing involves restraint, separation, and immobilization through tying the feet, which represent a source of acute stress, as documented by us in previous experiences [36]. Furthermore, cortisol release occurs even without shearing; the preparatory procedures for shearing are sufficient to determine a stressful stimulus causing hormone release from the adrenal cortex [3,37]. The absence of significant differences between shorn and unshorn animals within the same treatment group further suggests that the main stressors were associated with the preceding handling procedures, rather than the mechanical shearing process itself. In fact, simply isolating animals from the flock determines a marked stress with significant cortisol release [38]. Animals treated with melatonin showed lower cortisol levels compared to untreated animals at all sampling times, and treated animals, both shorn and unshorn, exhibited a smaller increase in cortisol compared to untreated groups, suggesting that melatonin was effective in attenuating both basal and acute stress-induced activation of the HPA axis.

This effect may be explained by the known interaction between melatonin and the circadian regulation of endocrine function. Melatonin and cortisol exhibit opposite daily rhythms, with melatonin peaking during the night and cortisol increasing in the early morning hours [39].

Melatonin has been shown to inhibit corticotropin-releasing hormone (CRH) and adrenocorticotropic hormone (ACTH) secretion, thereby reducing cortisol release [40]. The lower cortisol concentrations observed in treated animals likely reflect a combined central and peripheral inhibitory action of melatonin on stress-related neuroendocrine pathways. Melatonin receptors have been identified in key brain regions involved in stress regulation, including the hypothalamus and amygdala in sheep [41,42,43,44,45], suggesting a central modulatory role on the HPA axis. This is supported by our finding that melatonin-treated groups (A and B) showed consistently lower cortisol concentrations at all time points compared to untreated groups (C and D) (*p* < 0.05).

From a metabolic standpoint, stress-induced increases in glucose represent a physiological adaptation aimed at ensuring energy availability during acute challenges. The attenuated metabolic response observed in melatonin-treated animals suggests a reduced activation of stress-related metabolic pathways, likely secondary to a dampened HPA axis response. Glucose levels were higher in untreated groups compared to treated groups at all sampling times (*p* = 0.04). Peak cortisol and glucose levels occurred at T3 (approximately 15 min after the onset of procedures) in all groups, indicating a synchronized acute stress response that was quantitatively attenuated—but not abolished—by melatonin treatment. The absence of significant differences in triglyceride concentrations across all groups (*p* = 0.12) suggests that lipid metabolism was not substantially affected.

Regarding oxidative status, all measured parameters remained within the physiological range for the species [46]. However, melatonin-treated animals consistently showed a more favourable oxidative profile compared to untreated groups. Shearing induced a greater increase in ROMs in untreated animals, while treated animals displayed higher BAP and consequently a lower OSI (*p* = 0.02, *p* = 0.01, and *p* = 0.03, respectively). The improved oxidative profile in treated animals is consistent with the well-established antioxidant properties of melatonin, which acts as both a direct free radical scavenger and an inducer of endogenous antioxidant enzymes [47,48,49,50,51,52,53]. The fact that all groups showed an increase in ROMs and OSI and a decrease in BAP with the start of shearing procedures, but melatonin-treated groups consistently maintained a more favourable oxidative balance, suggests that melatonin’s dual mechanism of action—direct radical scavenging [48,49,50,51] and stimulation of endogenous antioxidant enzymes via its metabolites AFMK and AMK [53]—was effective under the acute stress conditions of this study.

The absence of significant differences between shorn and unshorn animals within the same treatment groups indicates that melatonin primarily modulates the overall stress response rather than the component specifically associated with the mechanical action of shearing. The widespread protective effect across different physiological parameters is consistent with melatonin’s ability to cross cell membranes and exert its action in all tissues and cellular organelles [52].

Melatonin is also produced in peripheral tissues and within mitochondria, where it acts as a local defence mechanism against oxidative stress [54,55,56,57]. This may help explain why treated animals maintained lower ROMs and OSI values despite acute stress, as well as lower glucose levels (*p* = 0.04).

Our results are also consistent with previous observations that in stressed animals, blood melatonin levels decrease despite constant pineal production [28], reflecting the body’s utilization of this indoleamine for neutralizing ROS [48].

Some limitations of the present study should be acknowledged. The study was conducted under field conditions during routine husbandry procedures, using animals available within the farm management schedule; accordingly, an a priori power calculation was not undertaken. In addition, behavioural and autonomic indicators of stress, as well as circulating melatonin concentrations, were not assessed. Furthermore, all blood samples were collected only in the morning (8:00 AM), which limits interpretation of circadian-dependent endocrine interactions. Therefore, no direct conclusions on animal welfare can be drawn in the absence of behavioural data. Future studies incorporating these parameters would allow a more comprehensive assessment of stress responses during shearing-related procedures.

## 5. Conclusions

The data further indicate that sheep shearing, including preparatory phases such as isolation and restraint, constitutes a significant stressor. Cortisol and glucose concentrations increased sharply at the onset of these procedures and remained elevated throughout the procedure. Melatonin-treated animals, whether shorn or not, exhibited a significantly attenuated cortisol response and lower glucose concentrations compared to untreated animals subjected to the same procedures. In addition, melatonin treatment was associated with an improved oxidative balance, both under resting condition and during the stress period, as evidenced by lower ROMs and OSI, and higher BAP.

In conclusion, melatonin administration effectively modulates the physiological response to stress by attenuating activation of the hypothalamic–pituitary–adrenal (HPA) axis and enhancing endogenous antioxidant defences. This dual action may contribute to reducing physiological stress associated with routine management procedures. The present findings suggest that the primary stressor is not related to shearing per se, but rather the associated handling procedures, particularly isolation and restraint. Consequently, efforts to improve animal welfare during shearing procedures should focus particularly on minimizing stress associated with handling, restraint, and isolation.

## Figures and Tables

**Table 1:** Mean ± s.d. values of cortisol (ng/dL) in the four groups of sheep during the four times of sampling.

Groups	T1	T2	T3	T4
A	10.4 ± 5.1 ^ax^	37.7 ± 12.6 ^ay^	48.1 ± 13.1 ^ay^	14.1 ± 7.4 ^ax^
B	11.2 ± 4.8 ^ax^	34.1 ± 11.3 ^ay^	42.6 ± 13.7 ^ay^	13.5 ± 8.4 ^ax^
C	14.3 ± 6.7 ^bx^	50.2 ± 15.7 ^by^	65.3 ± 16.8 ^by^	18.4 ± 8.2 ^bx^
D	15.7 ± 5.4 ^bx^	45.6 ± 14.4 ^by^	57.7 ± 17.2 ^by^	20.3 ± 8.5 ^bx^

A–D: experimental groups (A = melatonin-treated, shorn; B = melatonin-treated, unshorn; C = untreated, shorn; D = untreated, unshorn). T1–T4: sampling times (T1 = 24 h before shearing; T2 = shearing onset; T3 = post-shearing; T4 = 24 h after shearing, recovery). ^ab^ Mean values within a column with different superscript letters (a,b) differ significantly (*p* < 0.05) in sample comparison. ^xy^ Mean values within a row with different superscript symbols (x,y) differ significantly (*p* < 0.05) in group comparison.

**Table 2:** Mean ± s.d. values of glucose (mg/dL) in the 4 groups of sheep during the 4 times of sampling.

Groups	T1	T2	T3	T4
A	51.3 ± 12.9 ^ax^	78.4 ± 14.6 ^ay^	108.7 ± 26.2 ^az^	58.1 ± 14.2 ^ax^
B	54.7 ± 11.8 ^ax^	74.6 ± 15.2 ^ay^	112.5 ± 21.4 ^az^	55.7 ± 14.8 ^ax^
C	61.7 ± 14.4 ^bx^	98.6 ± 18.2 ^by^	127.5 ± 22.1 ^bz^	64.3 ± 13.6 ^bx^
D	63.5 ± 12.3 ^bx^	93.8 ± 22.7 ^by^	125.8 ± 19.6 ^bz^	68.3 ± 14.3 ^bx^

A–D: experimental groups (A = melatonin-treated, shorn; B = melatonin-treated, unshorn; C = untreated, shorn; D = untreated, unshorn). (T1 = 24 h before shearing; T2 = shearing onset; T3 = post-shearing; T4 = 24 h after shearing, recovery). ^ab^ Mean values within a column with different superscript letters (a,b) differ significantly (*p* < 0.05) in sample comparison. ^xyz^ Mean values within a row with different superscript symbols (x,y,z) differ significantly (*p* < 0.05) in group comparison.

**Table 3:** Mean ± s.d. values of triglycerides (mg/dL) in the four groups of sheep during the four times of sampling.

Groups	T1	T2	T3	T4
A	22.5 ± 5.5	26.7 ± 6.2	25.7 ± 5.8	21.5 ± 6.1
B	24.6 ± 5.8	25.1 ± 5.5	25.4 ± 4.9	23.6 ± 5.8
C	28.6 ± 6.4	29.7 ± 5.8	28.4 ± 5.4	26.8 ± 5.6
D	27.8 ± 5.9	30.4 ± 6.4	28.5 ± 6.5	27.7 ± 5.2

A–D: experimental groups (A = melatonin-treated, shorn; B = melatonin-treated, unshorn; C = untreated, shorn; D = untreated, unshorn). (T1 = 24 h before shearing; T2 = shearing onset; T3 = post-shearing; T4 = 24 h after shearing, recovery).

**Table 4:** Mean ± s.d. values of ROMs (U.Carr) in the four groups of sheep during the four times of sampling.

Groups	T1	T2	T3	T4
A	164.3 ± 42.7 ^ax^	210.7 ± 54.2 ^ax^	206.2 ± 52.7 ^ax^	179.6 ± 44.5 ^ax^
B	166.8 ± 45.3 ^ax^	200.5 ± 42.4 ^ax^	204.5 ± 40.6 ^ax^	165.3 ± 41.6 ^ax^
C	194.5 ± 47.3 ^by^	264.2 ± 56.1 ^by^	250.4 ± 54.5 ^by^	200.3 ± 50.8 ^by^
D	201.5 ± 44.1 ^by^	255.4 ± 47.3 ^by^	252.8 ± 52.6 ^by^	209.4 ± 49.3 ^by^

A–D: experimental groups (A = melatonin-treated, shorn; B = melatonin-treated, unshorn; C = untreated, shorn; D = untreated, unshorn). (T1 = 24 h before shearing; T2 = shearing onset; T3 = post-shearing; T4 = 24 h after shearing, recovery). ^ab^ Mean values within a column with different superscript letters (a,b) differ significantly (*p* < 0.05) in sample comparison. ^xy^ Mean values within a row with different superscript symbols (x,y) differ significantly (*p* < 0.05) in group comparison.

**Table 5:** Mean ± s.d. values of BAP (mEq/L) in the four groups of sheep during the four times of sampling.

Groups	T1	T2	T3	T4
A	2604.7 ± 527.4 ^ax^	2472.4 ± 487.7 ^ay^	2538.6 ± 501.8 ^ax^	2775.8 ± 551.3 ^ax^
B	2512.3 ± 548.3 ^ax^	2401.6 ± 584.1 ^ay^	2485.8 ± 519.8 ^ax^	2632.5 ± 555.8 ^ax^
C	2338.5 ± 583.2 ^bx^	2004.6 ± 617.4 ^by^	2098.2 ± 592.6 ^by^	2253.2 ± 532.5 ^bx^
D	2385.2 ± 542.7 ^bx^	1922.8 ± 510.4 ^by^	1987.8 ± 521.2 ^by^	2321.9 ± 486.5 ^bx^

A–D: experimental groups (A = melatonin-treated, shorn; B = melatonin-treated, unshorn; C = untreated, shorn; D = untreated, unshorn). (T1 = 24 h before shearing; T2 = shearing onset; T3 = post-shearing; T4 = 24 h after shearing, recovery). ^ab^ Mean values within a column with different superscript letters (a,b) differ significantly (*p* < 0.05) in sample comparison. ^xy^ Mean values within a row with different superscript symbols (x,y) differ significantly (*p* < 0.05) in group comparison.

**Table 6:** Mean ± s.d. values of OSI (ROMs/BAP × 100) in the four groups of sheep during the four times of sampling.

Groups	T1	T2	T3	T4
A	6.3 ± 0.3 ^ax^	8.5 ± 0.4 ^ay^	8.1 ± 0.3 ^ay^	6.4 ± 0.3 ^ax^
B	6.5 ± 0.3 ^ax^	8.7 ± 0.5 ^ay^	8.2 ± 0.3 ^ay^	6.6 ± 0.4 ^ax^
C	8.3 ± 0.4 ^bx^	13.2 ± 0.7 ^by^	11.9 ± 0.7 ^by^	8.8 ± 0.4 ^bx^
D	8.4 ± 0.4 ^bx^	13.3 ± 0.7 ^by^	12.7 ± 0.8 ^by^	8.7 ± 0.5 ^bx^

A–D: experimental groups (A = melatonin-treated, shorn; B = melatonin-treated, unshorn; C = untreated, shorn; D = untreated, unshorn). (T1 = 24 h before shearing; T2 = shearing onset; T3 = post-shearing; T4 = 24 h after shearing, recovery). ^ab^ Mean values within a column with different superscript letters (a,b) differ significantly (*p* < 0.05) in sample comparison. ^xy^ Mean values within a row with different superscript symbols (x,y) differ significantly (*p* < 0.05) in group comparison.

## Data Availability

The original contributions presented in this study are included in the article. Further inquiries can be directed at the corresponding author.

## Abbreviations

The following abbreviations are used in this manuscript:

ACTH	Adrenocorticotropic hormone
BAP	Biological antioxidant potential
BCS	Body condition score
CRH	Corticotropin-releasing hormone
DM	Dry matter
ELISA	Enzyme-linked immunosorbent assay
HPA	Hypothalamic–pituitary–adrenal
ME	Metabolizable energy
MT1	Melatonin receptor type 1
MT2	Melatonin receptor type 2
NEFA	Non-esterified fatty acids
OSI	Oxidative stress index
RNS	Reactive nitrogen species
ROMs	Reactive oxygen metabolites
ROS	Reactive oxygen species
